# The Relationship between Ambient Air Pollution and Heart Rate Variability Differs for Individuals with Heart and Pulmonary Disease

**DOI:** 10.1289/ehp.8337

**Published:** 2005-11-15

**Authors:** Amanda Wheeler, Antonella Zanobetti, Diane R. Gold, Joel Schwartz, Peter Stone, Helen H. Suh

**Affiliations:** 1 Health Canada, Ottawa, Ontario, Canada; 2 Department of Environmental Health, Harvard School of Public Health, Boston, Massachusetts, USA; 3 Channing Laboratory, Harvard Medical School, Brigham and Women’s Hospital, Boston, Massachusetts, USA; 4 Brigham and Women’s Hospital, Boston, Massachusetts, USA

**Keywords:** ambient pollution, chronic obstructive pulmonary disease, fine particulate matter, heart rate variability, myocardial infarctions, nitrogen dioxide

## Abstract

Associations between concentrations of ambient fine particles [particulate matter < 2.5 μ m aerodynamic diameter (PM_2.5_)] and heart rate variability (HRV) have differed by study population. We examined the effects of ambient pollution on HRV for 18 individuals with chronic obstructive pulmonary disease (COPD) and 12 individuals with recent myocardial infarction (MI) living in Atlanta, Georgia. HRV, baseline pulmonary function, and medication data were collected for each participant on 7 days in fall 1999 and/or spring 2000. Hourly ambient pollution concentrations were obtained from monitoring sites in Atlanta. The association between ambient pollution and HRV was examined using linear mixed-effect models. Ambient pollution had opposing effects on HRV in our COPD and MI participants, resulting in no significant effect of ambient pollution on HRV in the entire population for 1-, 4-, or 24-hr moving averages. For individuals with COPD, interquartile range (IQR) increases in 4-hr ambient PM_2.5_ (11.65 μ g/m^3^) and nitrogen dioxide (11.97 ppb) were associated with 8.3% [95% confidence interval (CI), 1.7–15.3%] and 7.7% (95% CI, 0.1–15.9%) increase in the SD of normal R-R intervals (SDNN), respectively. For individuals with MI, IQR increases in 4-hr PM_2.5_ (8.54 μ g/m^3^) and NO_2_ (9.25 ppb) were associated with a nonsignificant 2.9% (95% CI, –7.8 to 2.3) and significant 12.1 (95% CI, –19.5 to –4.0) decrease in SDNN. Beta-blocker and bronchodilator intake and baseline forced expiratory volume in 1 sec modified the PM–SDNN association significantly, with effects consistent with those by disease group. Results indicate heterogeneity in the autonomic response to air pollution due to differences in baseline health, with significant associations for ambient NO_2_ suggesting an important role for traffic-related pollution.

Recent epidemiologic studies have shown associations between ambient particle concentrations and changes in heart rate variability (HRV), a measure of autonomic function. In panel studies of healthy senior citizens (Creason et al. 2001; Gold et al. 2000), cardiac patients (Liao et al. 1999; Pope et al. 1999), and healthy adult boilermakers (Magari et al. 2001), airborne particles were associated with decreased HRV, a known risk factor for sudden death. Conflicting results were found in a study of seniors with chronic obstructive pulmonary disease (COPD), which showed no associations between HRV and particulate matter < 10 μ m aerodynamic diameter (PM_10_) and PM_2.5_ (Brauer et al. 2001), and in a study of healthy young highway patrolmen, which showed strong increases in HRV with PM_2.5_ exposures (Riediker et al. 2004). Results from these studies suggest that compromised autonomic control of the heart may play a role in the acute cardiovascular toxicity of particles but that this role may differ with the underlying health status of the individual. The impact of health status on the relationship between HRV and ambient PM, however, has not been examined directly, with panel studies conducted to date including participants of only one susceptible disease group. To examine this issue more directly, we conducted a study to evaluate associations between ambient fine particles (PM_2.5_) and HRV for sensitive individuals and examine whether these associations differed for individuals with preexisting pulmonary disease compared with those with cardiovascular disease.

## Materials and Methods

In fall 1999 and spring 2000, repeated health and exposure measurements were made under a protocol approved by the Harvard School of Public Health Human Subjects Committee. Measurements were made for individuals living in metropolitan Atlanta, Georgia, who had a myocardial infarction (MI) 3–12 months before the start of the study or had self-reported, physician-diagnosed moderate-to-severe COPD. Each of these individuals provided informed consent to participate in the study. Data for this analysis were collected as part of a more comprehensive investigation designed to examine the cardiovascular health effects of fine particles. Measurements were made for each participant over 7 consecutive days in one or both seasons, with health measurements made each morning and exposure measurements made each day, beginning 24 hr before the health measurements. Five participants were monitored simultaneously each 7-day period. Twenty-four and 22 individuals participated in the fall and spring, respectively, with 13 individuals (6 with MI, 7 with COPD) participating in both seasons. A total of 30 individuals (12 individuals with a recent MI and 18 individuals with COPD) participated in the study.

### Participant recruitment and baseline health assessment.

Participants were recruited from a number of locations in the metropolitan Atlanta area; the primary method for recruitment was through physician clinics and rehabilitation centers. Once the individual had expressed interest in participating and his or her primary care physician had granted approval, several screening procedures were undertaken. These included questionnaires on medical history, medication use, housing characteristics, and vitamin use; baseline spirometry; and a resting 12-lead electrocardiogram (ECG) (MAC6; Marquette Medical Systems Inc., Milwaukee, WI, USA). Exclusion criteria were similar to those of the Boston, Massachusetts, HRV study conducted by Gold et al. (2000) and included unstable angina, atrial flutter, atrial fibrillation, paced rhythm, left bundle branch block, on constant oxygen, smoking or living with smokers, and inability to walk on level ground. Two participants were unable to successfully complete the pulmonary function maneuver; as a result, baseline spirometry measurements were obtained for 28 of the 30 participants.

### Health monitoring.

Field technicians visited participants in their homes between 0700 hr and 1100 hr on each monitoring day. At the beginning of each visit, field technicians administered a questionnaire on health status, medication use, vitamin intake, housing characteristics, and activity patterns. Field technicians subsequently placed a Holter monitor (SEER MC ambulatory digital analysis recorder; GE Medical Systems, Milwaukee, WI, USA) on each participant and led the participant through a standardized HRV protocol.

The HRV protocol included continuous Holter monitoring using electrodes in a modified V5 and AVF position. The participant also wore a blood oximeter throughout the protocol (N-20P Oximeter; Nellcor, CA, USA). While wearing these devices, participants were asked to follow a standardized protocol (Gold et al. 2000) in which they were asked to rest in the supine position for 5 min, stand for 5 min, walk outside for 5 min, rest in the supine position for 5 min, and finally to perform paced breathing (5 sec inhalation and 5 sec exhalation) for 20 inhalation and exhalation cycles of 5 sec while being coached by a technician. The paced breathing portion of the protocol was designed to evaluate whether the effects of pollution on HRV were independent of respiratory rate, which might also be influenced by pollution levels, and to bring out vagal tone in the assessment of HRV. This standardized protocol has been used in other studies specifically for the evaluation of HRV (Sullivan 2005). During each of the 5-min protocol portions, respiratory rate was measured, followed by two blood pressure readings using an automated blood pressure machine (NIBP Vital Signs Monitor; Welch Allyn, Skaneateles Falls, NY, USA). The blood pressure readings during the standing portion of the protocol were taken after 2 min of standing had elapsed. After the outdoor exercise, the order of the blood pressure readings and respiratory rate was reversed.

We analyzed the Holter tapes using a Marquette MARS Workstation (Marquette Medical Systems, Milwaukee, WI, USA), with a sampling rate of 125 samples/sec, which is standard for routine HRV analyses and is “satisfactory” under the HRV guidelines (Task Force of the European Society of Cardiology and the North American Society of Pacing and Electrophysiology 1996). All analyses were reviewed by a trained technician for noise and artifact. Ectopic beats were excluded in these analyses, with a linearly interpolated QRS based on the prior beat’s R-R interval and the subsequent beat’s R-R interval inserted, as is standard. Holter tapes were analyzed for several measures of HRV, including both time and frequency domain outcomes. Time domain outcomes included the standard deviation of normal R-R intervals (SDNN), the square root of the mean of the sum of squares of differences between adjacent NN intervals (RMSSD), and the percentage of the absolute differences between successive normal R-R intervals that exceed 50 msec (PNN50). Frequency domain variables included high-frequency power of 0.15–0.40 Hz (HF) and low-frequency power of 0.04–0.15 Hz (LF).

A total of 300 Holter monitoring sessions were completed for 31 subjects, 28 of whom had five or more measurements. Of the 300 observations, 25 observations were excluded because of onset of atrial fibrillation, three because of second-degree block, and three were unreadable. One participant was subsequently excluded from all analyses because of atrial fibrillation. Of the 300 valid Holter sessions, three sessions were missing portions of the overall protocol. These sessions were not excluded from subsequent data analyses because results did not differ based on their inclusion in the model.

### Pollution measurements.

**Figure 1:**
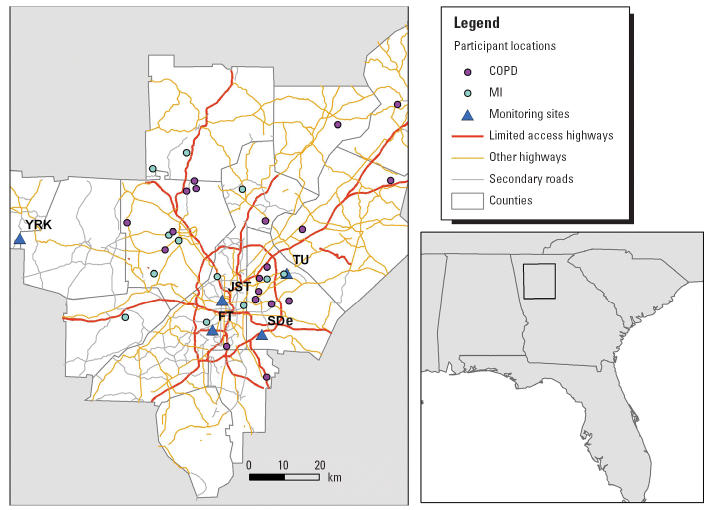
Locations of participants’ homes and the SAM sites, Atlanta, Georgia. Abbreviations: FT, Fort McPherson; JST, Jefferson St.; SDe, South Dekalb; TU, Tuckerville; YRK, Yorkville.

Hourly pollution and meteorologic data were obtained from sites located within the metropolitan Atlanta area that were operated as part of other studies (Figure 1). Hourly ambient PM_2.5_ concentrations were measured at the Yorkville, Fort McPherson, and Tucker stationary ambient monitoring (SAM) sites, which were operated as part of the Assessment of Spatial Aerosol Composition in Atlanta and Southeastern Aerosol Research and Characterization studies (National Research Council 1999). Sites were located throughout the metropolitan Atlanta area and were characterized by a variety of land uses. Tucker is classified as a suburban/commercial site, Yorkville as a rural site, and Fort McPherson as an urban site. Hourly PM_2.5_ concentrations were measured at these sites using a tapered element oscillating microbalance with a Nafion dryer placed upstream of the inlet (R&P, Albany, NY, USA). Hourly ozone concentrations were measured using methods approved by the U.S. Environmental Protection Agency (U.S. EPA 2004) at SAM sites located at Tucker, Yorkville, and South Dekalb, a suburban site, whereas hourly carbon monoxide, sulfur dioxide, and nitrogen dioxide concentrations were measured using U.S. EPA-approved methods (U.S. EPA 2004) at the Tucker and South Dekalb sites. Black carbon (BC) concentrations were measured using aethalometers at the Jefferson St. SAM site located in downtown Atlanta in a mixed industrial and residential site that was operated as part of the Aerosol and Inhalation Epidemiological Study network. Meteorologic data, including temperature and relative humidity, were also obtained from the Jefferson St. SAM site.

Analyses were conducted using three exposure periods: the hour during the recorded HRV protocol and the 4- and 24-hr mean before the HRV protocol. Calculated 4-and 24-hr mean exposures were considered valid when data for > 3 hr or > 18 hr (75% of the hours), respectively, were available. Because participant homes and SAM sites were located throughout metropolitan Atlanta, the average concentration for the available SAM sites was used as the PM_2.5_ and gaseous exposure measure. Exposure data were void if data were available from only one SAM site. Under these criteria, 100%, 84%, 89%, 91%, and 95% of the observations were valid for 4-hr exposures to PM_2.5_, NO_2_, CO, SO_2_, and O_3_, respectively.

### Data analysis.

We examined the association between ambient pollution and HRV (for the entire 35-min period) using linear mixed-effect models. The HRV outcomes measured in the models were log transformed. Models controlled for time, meteorologic, and subject-related variables, including random effects for subjects, and adjusting for body mass index (BMI), temperature, and relative humidity as continuous variables and sex, age, medication use, season, hour, and day of week as categorical variables. Medication use was included as a categorical variable for four medication types: beta-blockers, calcium channel blockers, angiotensin-converting enzyme (ACE) inhibitors, and bronchodilators (nonsteroidal long- and short-acting), with values based on whether the participant was or was not prescribed the medication. Because the relationship between weather and the outcome variables was nonlinear, both temperature and relative humidity were modeled using natural spline functions with 3 degrees of freedom. Models did not control for heart rate, which is inversely associated with HRV. When heart rate was included in the model, model results were similar, although with slightly lower coefficients.

To identify factors that modify the association between HRV and ambient pollution, parameters, including disease status, baseline pulmonary function, medication use, respiratory rate, air conditioning use, exercise during the protocol, BMI, age, and heart rate, were included separately in models. Medication use was controlled for in these models, with the exception of models examining effect modification by medication use. Because only individuals with COPD took bronchodilator medications, models that were stratified by disease status did not control for bronchodilator medications. Analyses of pollution and HRV were conducted with data combined across seasons. All statistical analyses were conducted using S-Plus (Insightful Corporation, Seattle, WA, USA). Unless otherwise indicated, statistical significance was determined based on a *p*-value of 0.05.

## Results

### Participant profiles.

Measurements were obtained for 30 individuals, 12 with recent MIs (3–6 months post-MI) and 18 with self-reported, physician-diagnosed moderate-to-severe COPD. In total, 265 observations were obtained over all participants, 159 for those with COPD and 106 for those with a recent MI. Participants lived throughout metropolitan Atlanta (Table 1). As shown in Table 1, the COPD participants were generally older, with a higher percentage of females compared with the MI cohort. As would be expected, the COPD cohort had lower baseline lung function [forced expiratory volume in 1 sec (FEV_1_)]. Medication use differed by disease group, with bronchodilators taken only by individuals with COPD and beta-blockers taken by a higher fraction of individuals with a recent MI. Fewer COPD participants were never-smokers.

### Ambient pollution concentrations.

Ambient pollution levels varied substantially across the monitoring period (Table 2), with similar distributions for the fall and spring monitoring seasons. Distributions of 1-hr (not shown) and 24-hr ambient pollution concentrations were comparable to corresponding 4-hr values. Four-hour PM_2.5_ concentrations were significantly correlated with 4-hr NO_2_ (*r* = 0.44, *p* < 0.001) and CO (*r* = 0.43, *p* < 0.001) concentrations in both seasons, with correlations strongest in the fall for both pollutants. In both seasons, 4-hr PM_2.5_ concentrations were also significantly correlated with corresponding elemental carbon (EC) levels (*r* = 0.51, *p* < 0.001). Correlations were also strong among 4-hr ambient EC, NO_2_, and CO concentrations (*r*_EC-NO2_ = 0.67, *p* < 0.001; *r*_EC-CO_ = 0.59, *p* < 0.001; *r*_NO2-CO_ = 0.46, *p* < 0.001), likely because motor vehicles are the major source for these pollutants outdoors.

For PM_2.5_, 4-hr mean concentrations were lowest at the rural Yorkville site (13.2 ± 7.6 μ g/m^3^) compared with the Tucker (21.9 ± 10.5 μ g/m^3^) and Fort McPherson (18.5 ± 9.1 μ g/m^3^) SAM sites. Despite these differences, their concentrations were strongly correlated, with Spearman correlation coefficients > 0.75 for comparisons among the SAM sites and > 0.89 for comparisons of the individual site concentrations with the mean concentration across the sites. Although mean 4-hr concentrations were comparable across sites, correlations among the sites’ 4-hr NO_2_ concentrations were more moderate but remained significant, with a Spearman correlation coefficient for a comparison of Tucker and South Dekalb concentrations of 0.44 and coefficients for comparisons with the mean NO_2_ concentration across the two sites > 0.72. Four-hour mean O_3_ concentrations differed substantially across the sites, with mean levels highest at Yorkville (33.8 ± 12.6 ppb), followed by Tucker (14.5 ± 10.5 ppb) and South Dekalb (8.0 ± 9.2 ppb) sites. Correlations among the sites were also weaker, primarily due to the low correlations observed for the rural Yorkville site (0.30 and 0.00 for Yorkville–Tucker and Yorkville–South Dekalb comparisons, respectively).

For EC, because hourly ambient EC concentrations were measured only at the Jefferson St. SAM site, spatial variability was evaluated using 24-hr ambient EC concentrations measured at the Yorkville and Jefferson St. SAM sites. The mean 24-hr concentration at Jefferson St. (1.81 ± 1.06 μ g/m^3^) was approximately twice that for YRK (0.77 ± 0.45 μ g/m^3^), which is consistent with the fact that Jefferson St. was located in an urban/suburban area and Yorkville was located in a rural area. Despite these differences in mean levels, 24-hr EC concentrations at these sites were moderately correlated during the study period, with a significant Spearman correlation of 0.47.

### HRV outcomes.

Several of the HRV outcomes varied significantly by disease group (Table 3). Individuals with COPD, for example, had significantly lower SDNN and LF values compared with individuals with a recent MI as determined using the Wilcoxon rank sum test (*p* = 0.012 and 0.0003, respectively). Heart rate, a potential modifier of HRV, was lower in the MI group (*p* < 0.0001), which may reflect beta-blocker use in the MI cohort. For all other outcomes, comparable values were found across disease group.

### Association between PM_2.5_ and HRV.

The association between ambient PM_2.5_ and SDNN was positive for individuals with COPD, with associations strongest for the 4-hr moving average (Table 4**)**. For the 4-hr moving average, SDNN rose by 7.5% (95% CI, 1.5–13.9%) per interquartile range (IQR) increase in ambient PM_2.5_ (10.64 μ g/m3). In comparison, opposing effects of ambient PM_2.5_ on HRV were found in individuals who were post-MI, resulting in no significant overall summary effect of ambient PM_2.5_ on HRV in the entire population for moving averages of 1-, 4-, or 24-hr (Table 4). When these analyses were performed using models that included an interaction term between PM_2.5_ and disease status, the disease group-specific effects of ambient PM_2.5_ on HRV were similar to those obtained from our stratified analyses, with a significant interaction term. As shown in Table 5, associations of PM_2.5_ and other HRV measures, RMSSD, PNN50, LF, HF, and LF:HF ratio, generally followed similar trends by disease status, with associations significant only for LF in individuals with COPD.

### Association between HRV and other pollutants.

Four-hour ambient NO_2_ concentrations were associated with changes in SDNN when analyses were stratified by disease status (Table 6), with results exhibiting trends consistent with those for ambient PM_2.5_. Effect sizes for NO_2_ were similar to those observed for ambient PM_2.5_ for individuals with COPD and were larger and more precise than those observed for ambient PM_2.5_ for individuals with an MI. An IQR increase in 4-hr NO_2_ concentrations (10.75 ppb) was associated with a 6.9% (95% CI, 0.1–14.2) increase in SDNN in individuals with COPD and a 13.9% (95% CI, –22.2 to –4.7) decrease in SDNN in persons with a recent MI. For EC, trends consistent with those for ambient PM_2.5_ and NO_2_ were observed; however, associations were insignificant. Ambient O_3_ concentrations were not associated with SDNN when data were examined for all subjects or were stratified by disease group (Table 6).

### Effect modification by additional diagnosis-related end points.

The effect of medication use, respiratory rate, baseline pulmonary function (as measured by FEV_1_), air conditioning use, exercise during HRV measurement, BMI, age, and heart rate on the association between 4-hr ambient pollution and overall SDNN was examined to determine whether these factors was responsible for the differences in response between the disease groups. Of these, medication use and baseline FEV_1_ were found to be significant effect modifiers for 4-hr PM_2.5_ and NO_2_ concentrations, with results comparable for the two pollutants (Table 7). Similar effect modification by medication use and baseline pulmonary function was also found for EC but with smaller effect sizes.

### Baseline FEV_1_.

Consistent with the results by disease group, baseline FEV_1_ (percent predicted) significantly modified the association between SDNN and 4-hr ambient PM_2.5_ and NO_2_ among all participants, with the PM_2.5_–SDNN association increasing with decreasing baseline FEV_1_. Coefficients for the main pollution effect and the FEV_1_ interaction term equaled 0.015 ± 0.004 (mean ± SE; *t*-value = 3.46) and –0.0002 ± 0.0001 (*t*-value = –3.18), respectively. For individuals with poor pulmonary function (baseline FEV_1_ 35% of predicted; 10th percentile for study population), these values corresponded to a 10.2% increase in SDNN for an IQR increase in 4-hr ambient PM_2.5_ concentrations, whereas for individuals with normal pulmonary function (baseline FEV_1_ 105% of predicted; 90th percentile for the study population), these values corresponded to a 2.5% decrease in SDNN for the same IQR increase in PM_2.5_ (Table 7).

### Medication use.

**Figure 2:**
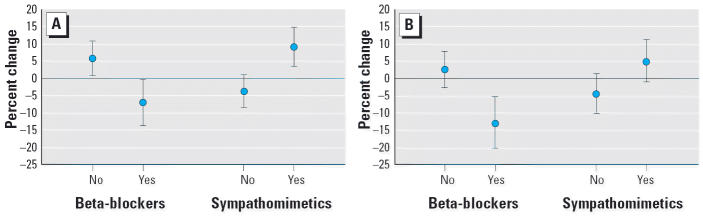
Percent change in SDNN with 4-hr PM_2.5_ (*A*) and NO_2_ (*B*), expressed with 95% CI per IQR change in PM_2.5_ (10.64 μ g/m^3^) or NO_2_ (10.75 ppb). Medication use status is based on whether participant took medication on the morning of the health measurement. All percent changes differed significantly by medication use at *p =* 0.05.

Beta-blocker and bronchodilator use on the health measurement day significantly modified the association between 4-hr ambient PM_2.5_ and NO_2_ concentrations and SDNN (Figure 2). SDNN decreased by 7.3% (95% CI, –13.8 to –0.35) for subjects taking beta-blockers and increased by 5.8% (95% CI, 0.91–10.8) for other subjects with an IQR increase in 4-hr ambient PM_2.5_. Responses were consistent in direction with those for individuals with an MI and COPD, respectively. Opposite patterns were observed for subjects taking bronchodilator medications, where positive SDNN–PM_2.5_ associations were found for those on the medication and negative associations were found for other subjects. The effect sizes for PM_2.5_-associated changes in SDNN for individuals taking medications were higher in magnitude compared with effect sizes for corresponding individuals with MI or COPD alone. Medications that were taken by individuals from both disease groups, including ACE inhibitors and calcium channel blockers, were not found to modify the SDNN–PM_2.5_ association significantly.

### Effect of spatial variability on effect estimates.

The effect of spatial variability in ambient PM_2.5_ on observed associations between overall SDNN and ambient PM_2.5_ and ambient NO_2_ was analyzed using data from each of the individual SAM sites. For PM_2.5_, spatial variability in ambient concentrations had little effect on the observed associations because both the magnitude and direction of the association between SDNN and 4-hr ambient PM_2.5_ were comparable across sites (Table 8**)**. These results are consistent with the strong correlations among the sites’ PM_2.5_ concentrations, with Spearman correlation coefficients > 0.87 for pairwise comparisons of the 4-hr concentrations at the individual sites with the 4-hr across-site mean concentrations. Results suggest that, for PM_2.5_, the mean ambient SAM PM_2.5_ concentration is a good indicator of ambient PM_2.5_ across the metropolitan Atlanta area.

For NO_2_, we found similar trends by disease status when associations were estimated using data for the individual SAM sites compared with the mean of these sites (Table 8). The magnitude and significance of the associations, however, dropped, with associations for individuals with MIs no longer significant when measurements at the South DeKalb site were used in the analysis. These findings suggest greater exposure error when measurements from single SAM sites were used to reflect exposures for our study population.

## Discussion

Findings from our study provide direct evidence of heterogeneity in the autonomic response to ambient pollution that is dependent on the underlying health status of the study population. Changes in HRV were significantly and positively associated with ambient PM_2.5_ concentrations for individuals with COPD. Although not statistically significant, observed associations were consistently negative for individuals with recent MI. Further support that the HRV response to ambient PM_2.5_ differs for individuals with MI and COPD was provided by the fact that we found comparable effect estimates, with significant differences between disease groups, using models that included an interaction term between pollution and disease status. Associations with ambient PM_2.5_ were strongest for the 4-hr moving average and for SDNN, an overall measure of HRV, although consistent trends with disease status were observed for other moving averages and other HRV measures, including RMSSD, HF, and the LF:HF ratio. We also observed strong and significant associations with SDNN by disease group with ambient NO_2_, and to a lesser extent with ambient EC. Because ambient NO_2_ and EC originate primarily from motor vehicles, our findings suggest that motor vehicle-related pollution may be partly responsible for the observed effects of ambient particles on HRV.

Because results were similar irrespective of whether models controlled for heart rate and because heart rate did not modify the PM_2.5_–HRV association, results suggest that the observed effect modification by disease status was due to factors other than differences in heart rate. For individuals with recent MI, the negative, albeit insignificant, association observed between ambient PM_2.5_ and HRV was consistent with previous studies of elderly individuals (Creason et al. 2001; Gold et al. 2000), elderly individuals with preexisting cardiovascular-related conditions (Liao et al. 1999), and occupationally exposed, middle-aged boilermakers (Magari et al. 2002).

In contrast, our finding of positive associations between PM_2.5_ and HRV for individuals with COPD is in direct contrast to findings from the only other published study of pollution effects on HRV in individuals with COPD (Brauer et al. 2001), in which HRV was measured repeatedly for 16 individuals with mild-to-moderate COPD (FEV_1_ ≥ 0.75 L) living in Vancouver, British Columbia, Canada. For these individuals, nonsignificant ambient PM_2.5_-associated decrements in SDNN and RMSSD were found, with the lack of statistical significance attributed to low ambient pollution levels and the small sample size. The negative direction of the PM_2.5_–HRV association in Vancouver was comparable in magnitude to that observed in elderly individuals living in Boston (Gold et al. 2000). The contrasting PM_2.5_ and HRV associations for the Vancouver (Brauer et al. 2001) and our COPD cohorts may result from the fact that the Vancouver cohort had less severe pulmonary disease compared with our cohort (as defined by the self-reported physician diagnoses). Positive associations between HRV and PM_2.5_ have been demonstrated previously in a study of a young, healthy cohort of highway patrolmen (Riedeker et al. 2004). For these healthy patrolmen, SDNN increased significantly by 11.7% per 10 μ g/m^3^ in PM_2.5_ exposures. This increase is comparable to our 7.5% increase for a similar change in ambient PM_2.5_ (10.64 μ g/m3). Positive associations for the patrolmen were attributed to increased vagal activity in the young and healthy cohort.

Cardiovascular autonomic neuropathy is common, although not universal, in patients with COPD (Chhabra 2005). Although many patients with COPD have marked baseline sympathetic activation (Heindl 2001), individuals with COPD have been demonstrated to respond to stimuli such as exercise with increased vagal tone as measured by increases in HF (Bartels et al. 2003). It is possible that ambient pollution has similar effects on increases in vagal tone among at least some subsets of patients with COPD.

Because our COPD participants were generally older and less active than our MI participants, it is difficult to determine whether the observed effect modification by disease group reflect differences in the participant characteristics or in the diseases themselves. Of the patient characteristics, medication use was an important modifier of the HRV–PM_2.5_ associations. Individuals with COPD and MI were generally prescribed different medications, with beta-blocker and bronchodilator use limited largely to individuals with a recent MI and COPD, respectively. Correspondingly, effect estimates for subjects taking beta-blockers exhibited similar trends as those for subjects with an MI, whereas effect estimates for subjects taking bronchodilators were consistent with those for individuals with COPD. As a result of this strong overlap between medication use and disease group, it is not possible to separate the effects of medication use from disease in this study.

Baseline pulmonary function, as measured using baseline FEV_1_, was also found to modify the association between ambient PM_2.5_ and SDNN. From our linear model estimating the modification of PM_2.5_ effects by level of FEV_1_, we estimate that the PM_2.5_–HRV association decreased significantly with increasing pulmonary function, comparable to a 10.2% increase in SDNN for an IQR change in ambient PM_2.5_ for individuals with poor pulmonary function (baseline FEV_1_ 35% of predicted) and a 2.5% decrease in SDNN for individuals with normal lung function (baseline FEV_1_ 105% of predicted). Because the baseline pulmonary function was normal and narrowly distributed for individuals with recent MI, the observed impact of baseline pulmonary function on the PM_2.5_–SDNN response largely reflects variation in baseline pulmonary function among individuals with COPD. Because the influence of baseline pulmonary function on HRV was not examined in previous studies, it is unknown whether baseline pulmonary function had similar impacts on the PM-associated HRV response in the previously reported studies. Evidence from cardiovascular health studies, however, suggests that respiratory health status may be an important modifier of the pollution-mediated autonomic response, because HRV in individuals with COPD has also been shown to decrease with FEV_1_ (Stein et al. 1998). Negative associations between average HRV and baseline FEV_1_, however, were not observed in our COPD cohort.

Results for PM_2.5_ were robust with respect to location of the SAM site because results were comparable when PM_2.5_ concentrations at individual SAM sites were used as the exposure measure, suggesting that spatial variation in PM_2.5_ was not a significant source of exposure error for our study participants. For NO_2_, similar sensitivity results show consistent trends in the NO_2_–HRV associations by disease group. Larger differences in the effect estimates, however, were found with different methods to estimate ambient NO_2_ exposures, suggesting that exposure error due to spatial variability was greater for NO_2_ compared with PM_2.5_. This greater exposure error is consistent with the fact that traffic, which varies spatially over short distances, is a significant source of outdoor NO_2_. Although comparable sensitivity analyses could not be conducted for BC, significant correlations between 24-hr concentrations measured at the Jefferson St. and Yorkville sites suggest that BC concentrations measured at the Jefferson St. SAM site were able to reflect BC concentrations across Atlanta. Because these correlations were more moderate compared with those for PM_2.5_ and were similar in magnitude to those for NO_2_, it is likely that spatial variation in outdoor BC levels contributed to error in our exposure estimates and thus may have affected our observed effect estimates. For both NO_2_ and BC, this greater exposure error may explain the lower and insignificant effect estimates found for these pollutants.

One important limitation of this investigation is the small number of individuals studied, with only 18 and 12 individuals with COPD and a recent MI, respectively. Our findings of effect modification by medication use and baseline pulmonary function, COPD, and a recent MI, however, were consistent with our findings of differential susceptibility for individuals with COPD and a recent MI. Further study is needed to identify factors responsible for this differential susceptibility and to identify whether the autonomic response of individuals with other health conditions also differ after ambient pollution exposures.

## Correction

The affiliation for D.R.G. was incorrect in the manuscript published online. It has been corrected here.

## Figures and Tables

**Table 1:** Participant characteristics by disease status.

	Disease group		
Characteristic	COPD	MI	All^a^
Sex^b^			
Male	7	10	30
Female	11	2	
Age (years)^c^			
Male	74 (71–76)	59 (49–70)	65 (55–73)
Female	64 (49–75)	69 (66–71)	
FEV_1_ (% predicted) c,d			
Male	67 (43–80)	99 (86–115)	69 (44–98)
Female	45 (36–59)	96 (91–101)	
BMI^c^	27 (22–31)	27 (25–28)	27 (25–29)
Medication use^a^			
Beta-blockers	1	8	9
Calcium channel blockers	4	3	7
ACE inhibitors	3	6	9
Bronchodilators, short acting^e^	12	0	12
Bronchodilator, long acting^f^	5	0	5

a Totals for all participants.

b Expressed as number of participants.

c Expressed as mean (interquartile range).

d Does not include data for two participants, for which baseline pulmonary function could not be measured.

e Albuterol, salmeterol, pirbuterol, ipratropium bromide and albuterol, and ipratropium bromide.

f Theophylline.

**Table 2:** Summary of meteorologic and air pollution levels.

			Percentile					
Parameter^a^	No.	Mean	10th	25th	50th	75th	90th	24 hr^b^
Meteorology (24 hr)								
Temperature (°C)	265	17.8	12.2	16.1	18.0	20.3	23.6	—
Relative humidity (%)	265	70.3	52.5	57.4	70.1	82.6	92.6	—
Pollution (4 hr)								
PM_2.5_ (μ g/m^3^)	264	17.8	7.0	11.6	16.5	22.2	30.9	17.2
EC (μ g/m^3^)	260	2.3	0.9	1.2	1.6	2.9	4.4	2.0
O_3_ (ppb)	251	18.5	9.0	12.7	17.0	22.5	30.3	29.4
CO (ppb)	236	362.0	169.7	221.5	304.3	398.1	682.1	332.1
SO_2_ (ppb)	240	1.9	0.7	0.9	1.5	2.4	3.6	2.5
NO_2_ (ppb)	222	17.8	7.1	12.0	17.0	22.8	29.8	17.1

a Values correspond to the mean for the 24-hr or 4-hr periods before the HRV measurements.

b Corresponding 24-hr mean values for time period before the health measurements.

**Table 3:** HRV [mean (10th percentile, 90th percentile)] by disease status.^a^

HRV parameter	COPD (*n* = 18)	MI (*n* = 12)
SDNN (msec)	104.4 (55.2, 186.2)	114.8 (63.8, 207.4)
RMSSD (msec)	44.5 (14.4, 129.4)	39.2 (12.6, 115.2)
PNN50 (%)	10.0 (0.4, 30.9)	6.4 (0.2, 23.2)
LF (msec^2^)	759.1 (62.8, 3700.6)	808.1 (115.5, 2726.2)
HF (msec^2^)	333.4 (36.1, 1278.1)	361.0 (49.8, 1356.9)
LF:HF ratio	2.5 (1.1, 6.8)	3.7 (0.9, 7.4)
Heart rate (bpm)	80.9 (64.2, 105.7)	68.6 (54.0, 86.7)

a Includes data for all participants in both seasons; means and 10th and 90th percentiles expressed as the values for the participant means.

**Table 4:** Association between ambient PM_2.5_ and overall SDNN.

PM_2.5_ integration period	IQR (μ g/m^3^)	Percent change	95% CI
1 hr	10.3	1.50	–2.22 to 5.36
MI only	9.74	–2.75	–7.93 to 2.72
COPD only	10.66	5.07*	0.01 to 10.38*
4 hr	10.63	1.97	–2.30 to 6.43
MI only	8.54	–2.89	–7.79 to 2.27
COPD only	11.65	8.29*	1.71 to 15.30*
24 hr	8.00	–1.00	–4.67 to 2.82
MI only	8.05	–4.38	–9.42 to 0.94
COPD only	7.84	1.58	–3.59 to 7.01

Percent change in overall SDNN (with 95% CIs) expressed per IQR change in ambient PM_2.5_. Model does not include heart rate.

* Statistically significant estimate.

**Table 5:** Association between ambient 4-hr PM_2.5_ and other HRV parameters.

HRV measure	Percent change	95% CI	*t*-Value
SDNN	1.97	–2.30 to 6.43	0.90
MI	–2.89	1.71 to 2.27	–1.11
COPD	8.29*	–7.79 to 15.30	2.49
RMSSD	–0.92	–10.19 to 9.30	–0.18
MI	–11.38	–23.03 to 2.03	–1.68
COPD	9.77	–5.37 to 27.35	1.23
PNN50	–2.96	–18.15 to 15.03	–0.35
MI	–10.18	–29.94 to 15.17	–0.85
COPD	–1.19	–21.07 to 23.70	0.10
LF	14.04	–1.84 to 32.49	1.72
MI	1.12	–15.52 to 21.03	0.12
COPD	35.88*	6.79 to 72.90	2.49
HF	5.03	–9.96 to 22.52	0.62
MI	–6.48	–25.68 to 17.70	–0.57
COPD	19.41	–4.05 to 48.59	1.59
LF:HF ratio	1.85	–4.91 to 9.09	0.52
MI	–1.28	–9.16 to 7.28	–0.30
COPD	6.68	–4.02 to 18.59	1.20

Data are percent change in HRV (overall protocol) expressed per IQR change in 4-hr ambient PM_2.5._ IQR changes were 10.6, 8.5, and 11.7 μ g/m^3^ for the 4-hr mean of the SAM sites for all, MI, and COPD participants, respectively. Model does not include heart rate.

* Statistically significant estimate.

**Table 6:** Association between 4-hr EC, NO_2_, and O_3_ concentrations and overall SDNN.

Pollutant	IQR	Percent change	95% CI
EC	1.68	–0.83	–3.27 to 1.67
MI only	1.39	–1.06	–4.19 to 2.16
COPD only	1.92	0.51	–3.33 to 4.51
NO_2_	10.66	–0.49	–5.4 to 4.6
MI only	9.25	–12.09*	–19.5 to –4.0
COPD only	11.97	7.70*	0.1 to 15.9
O_3_	9.61	0.75	–3.6 to 5.3
MI only	8.08	0.13	–6.5 to 7.2
COPD only	10.66	2.45	–3.4 to 8.7

Data are percent change in overall SDNN (with 95% CIs) expressed per IQR change in pollutant concentrations. Units for IQR are μ g/m^3^ for EC, and ppb for O_3_ and NO_2_. Model does not include heart rate.

* Statistically significant estimate.

**Table 7:** Association of SDNN and ambient 4-hr concentrations: baseline FEV_1_ (percent predicted) as an effect modifier.

Pollutant	FEV_1_	Percent change	95% CI^a^
PM_2.5_ (10.6 μ g/m^3^)	105	–2.5	–7.8 to 3.2
	35	10.2*	3.8 to 17.0
EC (1.7 μ g/m^3^)	105	–3.9*	–7.4 to –0.3
	35	2.5	–1.3 to 6.4
NO_2_ (10.7 ppb)	105	–5.4	–12.0 to 1.8
	35	5.7	–1.2 to 12.9
O_3_ (9.6 ppb)	105	–0.4	–7.1 to 6.8
	35	1.1	–4.1 to 6.6

a Expressed as a change per IQR for the 4-hr mean ambient concentration.

* Statistically significant association.

**Table 8:** Association between ambient 4-hr PM_2.5_ and SDNN by SAM site.

Pollutant/SAM site	IQR	Percent change	95% CI	*t*-Value
PM_2.5_				
Mean of SAM sites	10.63	1.97	–2.30 to 6.43	0.90
MI	8.54	–2.89	–7.79 to 2.27	–1.11
COPD	11.65	8.29*	1.71 to 15.30	2.49
Tucker	15.40	1.74	–3.2 to 6.9	0.68
MI	14.33	–3.59	–10.3 to 3.6	–1.00
COPD	15.98	7.39*	0.4 to 14.9	2.07
Fort McPherson	13.04	1.13	–3.7 to 6.2	0.45
MI	12.68	–5.35	–11.7 to 1.5	–1.55
COPD	13.78	7.10	0.05 to 14.7	1.97
Yorkville	8.35	2.71	–1.3 to 6.9	1.31
MI	7.99	–3.63	–9.0 to 2.0	–1.27
COPD	8.52	8.23*	2.2 to 14.6	2.72
NO_2_				
Mean of SAM sites	10.66	–0.49	–5.4 to 4.7	–0.19
MI	9.25	–13.88*	–22.1 to –4.7	–2.88
COPD	11.97	6.89*	0.1 to 14.2	1.97
Tucker	13.75	–1.61	–5.9 to 2.9	–0.71
MI	12.88	–10.36*	–17.7 to –2.4	–2.53
COPD	13.75	3.75	–2.1 to 10.0	1.24
South Dekalb	12.06	0.57	–4.7 to 6.1	0.21
MI	11.06	–6.50	–14.6 to 2.4	–1.45
COPD	12.19	4.99	–1.7 to 12.2	1.44

Data are percent change in overall SDNN expressed per IQR change in ambient PM_2.5_. Model does not include heart rate.

* Statistically significant estimate.
